# ROR2, a driver of “phenotype switching” in melanoma?

**DOI:** 10.1186/s12935-022-02711-x

**Published:** 2022-09-20

**Authors:** Pablo Lopez-Bergami

**Affiliations:** 1 Centro de Estudios Biomédicos, Básicos, Aplicados y Desarrollo (CEBBAD), Universidad Maimónides, Hidalgo 775, 6th Floor, Lab 602, 1405 Buenos Aires, Argentina; 2 Consejo Nacional de Investigaciones Científicas y Técnicas (CONICET), 1425 Buenos Aires, Argentina

**Keywords:** ROR2, Melanoma, Phenotype switching, Epithelial to mesenchymal transition, Cell plasticity

## Abstract

Receptor tyrosine kinase-like orphan receptor 2 (ROR2) is a receptor for the Wnt5a ligand that was shown to play a dual role in cancer. ROR2 was shown to either suppress or promote tumor progression in different tumor types by regulating the same biological processes (i.e. proliferation, invasion) in opposite ways. We have recently observed that ROR2 plays multiple and somewhat contradictory roles in melanoma where it impairs cell proliferation but promotes migration, EMT and chemoresistance. In the present article, ROR2 is proposed to be a major driver of “phenotype switching” in melanoma that can tilt the cellular behavior toward proliferative or invasive phenotypes. This function of ROR2 has therapeutic implications since it would provide an opportunity for targeting specific phenotypes such as invasive and drug-resistant ones by inhibiting ROR2.

## Introduction

Receptor tyrosine kinase-like orphan receptor 2 (ROR2) is a receptor for the Wnt5a ligand that plays a major role during embryonic development. ROR2 expression is strongly downregulated after birth but was shown to express aberrantly in cancer as well as in other pathological conditions in the adult. In cancer, ROR2 possesses a dual role by either suppressing or promoting tumor progression in different tumor types [1, 2]. For example, ROR2 has a tumor-suppressive function in endometrial and colon cancer, in medulloblastoma, and hepatocellular and gastric carcinoma. In several other tumor types including cancers from the breast, prostate, and ovary among others (reviewed in [2]), ROR2 promotes cancer. The underlying reasons for this discrepancy have not been elucidated and ROR2 has been considered alternatively an oncogene or a tumor suppressor gene depending on the tumor type. Interestingly, ROR2 exerts these dual functions by regulating the same biological processes in opposite ways. For instance, the anti- and pro-tumoral function of ROR2 have been associated with the inhibition or stimulation of cell proliferation, respectively. Similarly, ROR2 was shown to either enhance or inhibit cell migration and invasion [2].

## Main text

**Fig. 1:**
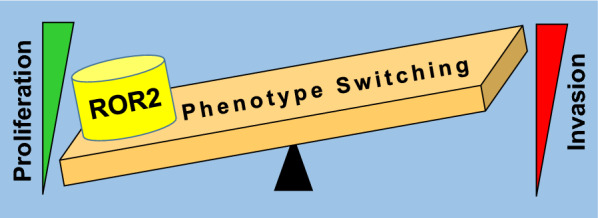
ROR2 is proposed to act as a driver of “phenotype switching” in melanoma. Increased ROR2 levels promotes a switch from a highly proliferative, less invasive state to a highly invasive, less proliferative state

Recent findings from our group have established that ROR2 can play this dual role simultaneously in melanoma cell lines. Unlike what was described in other cancers, the dual role of ROR2 in melanoma is associated with the regulation of different biological processes. We have shown that ROR2 impairs cell cycle progression and consequently inhibits the proliferation of melanoma [3]. However, ROR2 also promotes migration, epithelial to mesenchymal transition (EMT), and chemoresistance in the same cell lines [4, 5]. At the molecular level, we have determined that the anti-proliferative function depends on the inhibition of the PI3K/Akt pathway [4] whereas the pro-tumoral effect is linked to the activation of the MAPK/ERK pathway [5]. These findings make it difficult to classify ROR2 as either an oncogene or a tumor suppressor gene in melanoma. Instead, the function of ROR2 can be better understood if viewed as a regulator of “phenotype switching” (Fig. 1). This term was coined to describe the uncoupling of proliferation and invasion in melanoma and the reversible transition of melanoma cells from a highly proliferative, less invasive state to a highly invasive, less proliferative state regulated by both intrinsic and extrinsic signals [6]. Thus, similar to the other few proteins identified as regulators of “phenotype switching” (i.e. MITF, PHF19, BRN2, BORIS/CTCFL [6–9]), ROR2 can tilt the cellular behavior toward proliferation or invasion. Indeed, decreased ROR2 levels allow a high proliferative/low invasive state whereas high ROR2 levels promote a low proliferative/high invasive state (Fig. 1). Interestingly, a few years ago, it was shown that ROR2 expression was upregulated during phenotypic plasticity induced by hypoxia and that its expression correlated with the invasive phenotype [10]. The recent observations suggest a more important role for ROR2, by behaving as an inducer of “phenotype switching” in normoxia and not just as a marker of this process under hypoxia. By assuming the proposed role, ROR2 would have a dual impact on melanoma. On one side, ROR2 expression would slow down tumor growth but on the other hand, it would favor an invasive phenotype that facilitates metastasis. Since both proliferation and invasion are critical at different disease stages, ROR2 would play different roles in melanoma initiation, promotion, and progression. This reveals an important difference with respect to the role of Wnt5a, the cognate ROR2 ligand, which has long been implicated in melanoma progression and metastasis [11]. Unlike ROR2, Wnt5a also promotes proliferation [12], thus behaving as a classical oncogene. These observations seem to suggest that ROR2 also has Wnt5a-independent functions.

For these drastic phenotypic changes to occur, a significant gene expression reprogramming is likely to take place. Along this line, all the proteins associated thus far with the triggering of “phenotype switching” are directly implicated in the regulation of gene transcription. For example, MITF and BRN2 are transcription factors, BORIS is a transcriptional modulator and PHF19 is an epigenetic transcriptional repressor. In contrast, the biochemical function of ROR2, a tyrosine kinase receptor (TKR), is completely different, a fact that can be viewed as an impediment to regulating “phenotype switching”. However, similar to other TKR important in melanoma such as AXL and EGFR [13], ROR2 does alter the expression of numerous proteins in melanoma [3–5] and other cancers [14]. Although the precise mechanism has not been completely elucidated, the signal triggered by aberrant ROR2 expression can be transduced by different signaling pathways and modulate transcription factor activity (i.e. AP-1, NFAT, etc.) [2]. Moreover, several transcription factors critical for “phenotype switching” such as Snail, ZEB1, Twist, and Slug [15] were shown to be upregulated by ROR2 [5]. In agreement with this mechanism, the upregulation of Snail induced by ROR2 was blocked by MAPK inhibitors [5]. Alternatively, since ROR receptors have been shown to translocate to the nucleus [16, 17], it is possible to speculate that ROR2 can regulate gene expression by directly interacting with transcription factors, as previously demonstrated by many TKRs [18] including its sister receptor ROR1 [19]. Up to the present, studies on the function of nuclear ROR2 are limited.

The switch from proliferative to invasive phenotypes is of great relevance in cancer in general since similar transitions have been observed in other tumor types. The best know example is EMT described in epithelial tumors, a process in which epithelial cells lose their epithelial characteristics and gain mesenchymal features [20]. Moreover, “phenotype switching” and EMT share many features. For example, both processes are characterized by a transition between poorly and highly invasive phenotypes, both can be initiated by signals from the microenvironment such as hypoxia, TGFβ, WNTs, and Notch, and both can take place both during embryonic development and cancer progression [21]. Given these similitudes, tracing parallelisms between melanoma “phenotype switch” and EMT could help to improve our understanding of the progression of epithelial cancers. However, an obstacle to this is that some “phenotype switching” inducers like MITF and BRN2 express poorly in other cancers, and others such as BORIS/CTCFL and PHF19 have been scarcely studied in epithelial cancers. In contrast, the expression of ROR2 and its participation in several tumor types have been more extensively documented [1, 2]. Therefore, further studies on ROR2 function under the lens of its role in “phenotype switching” might contribute to a better understanding of cancer progression.

Given the association of ROR2 with cancer and its relatively restricted expression in the cell surface of tumor cells, targeting ROR2 (particularly using monoclonal antibodies) is being evaluated as a new therapy against cancer [2]. “Phenotype switching” has been proposed as a major contributor to melanoma tumor heterogeneity, a key determinant of the emergence of therapy-resistant cells, and a major cause of treatment failure and tumor recurrence. Thus, “drugging” ROR2 would provide the opportunity for specifically targeting invasive cells associated with the dissemination of cancer. Similar to epithelial cancer cells undergoing EMT [22], melanoma cells switched to the invasive phenotype are associated with greater resistance to therapy [23]. Therefore anti-ROR2 therapies might also target these drug-resistant phenotypes. Hence, the characterization of the signaling events regulating phenotype plasticity contributes to developing combination therapies that target multiple subpopulations of tumor cells and control tumor cells for longer periods. However, caution should be exercised when using ROR2-based therapies since they might be detrimental in some contexts, such as in those cancers where ROR2 behaves more clearly like a tumor suppressor gene. Even in melanoma, targeting ROR2 might provide a greater proliferative capacity to melanoma cells that already spread to secondary sites, thus increasing the tumor burden.

## Outlook

Over the years, basic studies have revealed the association of dozens of proteins with melanoma progression. However, very few of them were shown to simultaneously regulate proliferation and invasion in opposite directions. So, further elucidation of the role of ROR2 in “phenotype switching” will contribute to a better understanding of this process and will provide interesting new alternatives for cancer treatment.

## Data Availability

Not applicable.
